# Variability of flow-mediated dilation across lower and upper limb conduit arteries

**DOI:** 10.1007/s00421-024-05517-z

**Published:** 2024-06-15

**Authors:** Alessio Daniele, Samuel J. E. Lucas, Catarina Rendeiro

**Affiliations:** 1 School of Sport, Exercise and Rehabilitation Sciences, University of Birmingham, Birmingham, B15 2TT UK; 2 Centre for Human Brain Health, University of Birmingham, Birmingham, UK

**Keywords:** Flow-mediated dilation, Brachial, Femoral, Endothelial, Repeatability

## Abstract

Endothelial dysfunction is an early predictor of atherosclerosis and cardiovascular disease. Flow-mediated dilation (FMD) is the gold standard to assess endothelial function in humans. FMD reproducibility has been mainly assessed in the brachial artery (BA) with limited research in lower limb arteries. The purpose of this study was to compare FMD reproducibility in the upper limb BA and lower limb superficial femoral artery (SFA) in young healthy adults.

Fifteen young healthy adults (nine males; six females) underwent FMD, resting diameter, velocity, and shear rate measurements on three occasions to determine intra-and inter-day reproducibility in both BA and SFA, assessed by coefficient of variation (CV), intraclass correlation coefficient (ICC), and Bland–Altman plots.

BA FMD CVs (intra-day: 4.2%; inter-day: 8.7%) and ICCs (intra-day: 0.967; inter-day: 0.903) indicated excellent reproducibility and reliability, while for SFA FMD, both CVs (intra-day: 11.6%; inter-day: 26.7%) and ICCs (intra-day: 0.898; inter-day: 0.651) showed good/moderate reproducibility and reliability. BA FMD was significantly more reproducible than SFA FMD (*p* < 0.05). Diameter reproducibility was excellent and similar between arteries, while resting velocity and shear rate have lower reproducibility in the BA compared to SFA. Bland–Altman plots displayed no proportional and fixed bias between measurements.

In summary, SFA FMD is less reproducible than BA FMD, with identical volume of ultrasound training. Given the increasing interest in using SFA FMD to test the efficacy of interventions targeting lower limb’s vascular health and as a potential biomarker for peripheral arterial disease risk, future studies should ensure higher levels of training for adequate reproducibility.

**Supplementary Information:**

The online version contains supplementary material available at 10.1007/s00421-024-05517-z.

## Introduction

Detrimental alterations of normal endothelial physiology, more commonly referred to as endothelial dysfunction, represents an early predictor of different pathologies including atherosclerosis and cardiovascular disease (CVD) (Jay Widmer and Lerman 2014; Mudau et al. 2012). The vascular endothelium is a monolayer of endothelial cells that constitute the innermost lining of the blood vessel wall (Félétou 2011; Krüger-Genge et al. 2019), playing a crucial role in regulating blood flow, platelet aggregation, hemostasis, and angiogenesis (Félétou 2011; Rajendran et al. 2013; Yau et al. 2015). More importantly, endothelial cells are involved in the regulation of vascular tone via the release of vasodilatory (e.g., nitric oxide [NO]) and vasoconstrictive factors (e.g., endothelin-1) (Sandoo et al. 2010).

Endothelial function can be assessed using a non-invasive technique known as flow-mediated dilation (FMD). FMD was first introduced in 1992 (Celermajer et al. 1992) and is now widely used, mainly in scientific research (Mućka et al. 2022), to quantify NO-dependent endothelial function of peripheral conduit arteries in humans (Thijssen et al. 2019). Peripheral conduit arteries such as the brachial (Green et al. 2014) and superficial femoral artery (SFA) (Kooijman et al. 2008) have been demonstrated to be largely mediated by NO, therefore reflecting endothelium-dependent vascular function. A decline in FMD reflects endothelial dysfunction, which is typically an early sign of atherosclerosis (Mudau et al. 2012; Thijssen et al. 2019). Most importantly, FMD of the brachial artery (BA) is reflective of future risk of CVD, with a decline of 1% in FMD being associated with an increased risk of a future CVD event (of up to 13%) (Inaba et al. 2010). Differently, lower limb FMD seems to be more specifically associated with risk of lower limb atherosclerosis and peripheral arterial disease (PAD) (Heinen et al. 2015). More recently, it has been shown that both brachial and femoral FMDs decline with age to the same extent (Bapir et al. 2022b).

Briefly, the FMD protocol requires the use of an ultrasound device (ideally, high-resolution, and duplex), to image a post-ischemic increase in arterial blood flow (and therefore shear stress) that results in a transient increase in diameter (i.e., vasodilation) of the imaged conduit artery (Thijssen et al. 2019). Reproducibility of this technique has been a matter of investigation due to substantial variations in several aspects of the protocol. These include mainly the sonographer’s skills (during the protocol and video analysis), the ultrasound equipment (e.g., with or without the use of a stereotactic adjustable probe-holding tool), the wall tracking system during video analysis (e.g., manual vs. automatic edge-detection system), and cuff placement (e.g., distal vs. proximal positioning). Thus far, despite specific guidelines being published with the aim of standardizing the FMD protocol (Harris et al. 2010; Thijssen et al. 2011, 2019), not all investigators in the scientific community closely follow them. Representing an issue of considerable importance, the lack of strict adherence to FMD guidelines limits the ability to directly compare data sets generated by independent laboratories and reproduce research outcomes. Several reproducibility studies in brachial FMD report coefficients of variation (CV) of more than 9% and 14% for intra-day and inter-day, respectively (e.g., Charakida et al. 2013; Craiem et al. 2007). More importantly, the vast majority of these studies investigated FMD reproducibility exclusively on the BA, with currently only a few studies focusing on lower limb FMD reproducibility, in the common femoral (Bapir et al. 2022b; Ratcliffe et al. 2017) and popliteal arteries (McLay et al. 2016). However, a direct comparison of intra-and inter-day reproducibility between brachial and femoral arteries has not been formally investigated. Therefore, the extent to which FMD reproducibility and reliability in the lower limb arteries are comparable to those of the brachial, assuming a similar volume of training, remains largely unexplored. This is particularly important because additional technical challenges arise when assessing FMD in the lower limbs (e.g., artery accessibility, volunteer discomfort, stability of the ultrasound probe during the ischemic challenge), which might result in poorer reproducibility and/or higher training needed to achieve lower methodological errors. Furthermore, estimating FMD reproducibility in the lower limb arteries is highly critical, given that the technique is currently being explored as a promising biomarker of lower extremity vascular health for PAD risk, prevention and treatment (Heinen et al. 2015). A recent systematic review has reported that the worldwide prevalence of PAD in adults aged 25 years and older was more than 5% (equivalent to more than 230 million people) (Song et al. 2019). PAD may negatively affect quality of life (Dumville et al. 2004) and may lead to serious health complications such as myocardial infarction, vascular dementia, and intermittent claudication (Morley et al. 2018). As lower limb FMD is likely to become an important diagnostic tool in the clinical setting, it is critical to clearly establish its reliability as a measure. More recently, it has also been used in more applied fields to assess the effects of physical inactivity, exercise, or nutritional supplementations on lower limb endothelial function (Bapir et al. 2022a; Daniele et al. 2022; Fuertes-Kenneally et al. 2023; Walker et al. 2019). Therefore, the present study compared, for the first time, intra-day and inter-day FMD reproducibility and reliability in the BA and SFA in young healthy adults, as well as, resting arterial diameter, blood velocity, and shear rate reproducibility, given that these parameters might contribute to FMD variability.

## Materials and methods

### Ethical approval

The study was approved by the University of Birmingham Ethics Committee (ERN_18-1707). Informed written consent was obtained from all participants before enrolment in the study.

### Participants

Young healthy adults, both males and females (aged 18–45 years old) were recruited from the University of Birmingham (Birmingham, England) and surrounding community. Prior to the participation in the study, all participants provided a signed informed consent, and completed a general health and lifestyle questionnaire. Participants were required to visit the Vascular Function Laboratory at the School of Sport, Exercise and Rehabilitation Sciences at the University of Birmingham on three occasions. Prior to each experimental session, participants were asked to refrain from caffeine-containing beverages for at least 12 h, and alcoholic beverages and any form of physical activity (above light intensity) for at least 24 h. Individuals with a history or symptoms of cardiovascular, renal, pulmonary, metabolic, or neurologic disease, hypertension (blood pressure higher than 140/90 mmHg), diabetes mellitus, anemia, asthma, immune conditions, or high cholesterol were excluded from the study. In addition, smokers, individuals who were on weight-reducing diets or using prescribed/over-the-counter medications, or had recently undergone prolonged bed-rest periods, were also excluded from the study.

### Experimental procedures

**Fig. 1:**
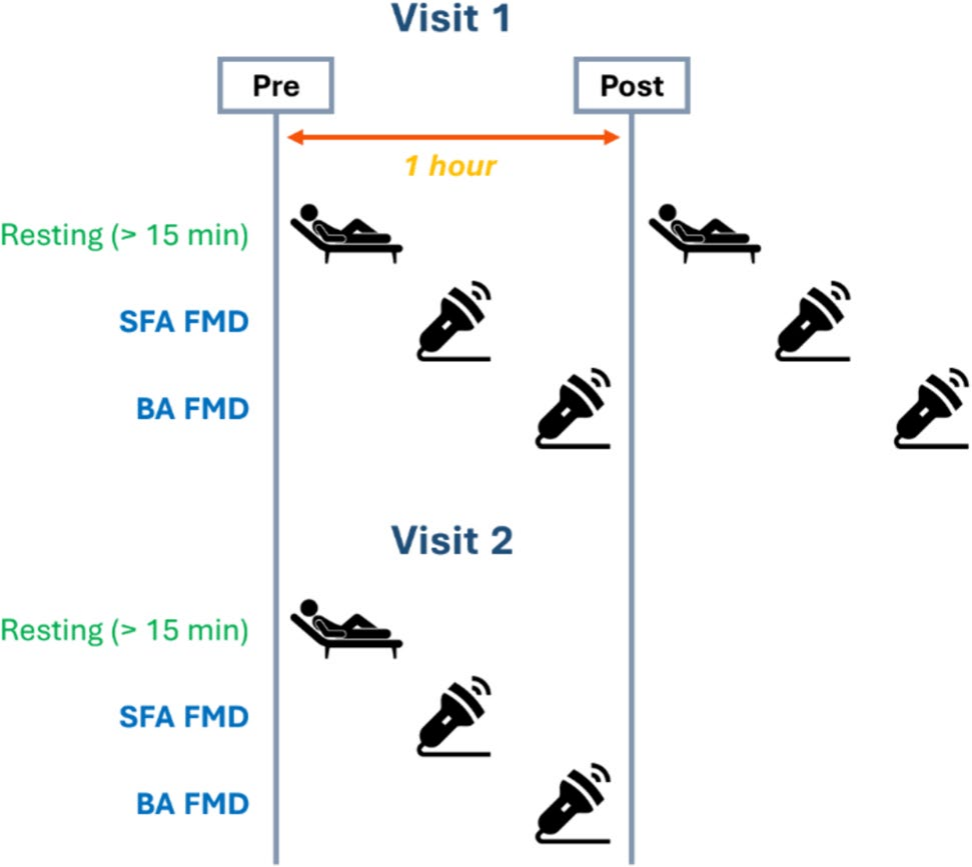
Schematic representation of the study design, showing the FMD measurements taken during visit 1 and visit 2. *BA, brachial artery; FMD, flow-mediated dilation; SFA, superficial femoral artery*

A schematic representation of the study design is presented in Fig. 1.

The study consisted of three study visits (familiarization visit, visit 1, and visit 2). A familiarization visit was performed prior to visit 1 to allow participants to familiarize themselves with the ultrasound measures, and for the researcher to localize both femoral and brachial arteries and undergo practice FMD protocols. No participants were excluded following the familiarization visit. Visit 1 and 2 were separated by approximately 7 days in male participants and performed within 2 consecutive days for female participants, to minimize the effects of their monthly hormonal variation on FMD outcomes (Brandão et al. 2014; Hashimoto et al. 1995). As recommended in contemporary FMD guidelines (Harris et al. 2010; Thijssen et al. 2019), all study visits were performed in a quiet, darkened and temperature-controlled laboratory (22–24 °C). Upon arrival, participants rested in supine position on a horizontal bed for at least 15 min. Afterward, while still in supine position, FMD of the SFA and BA were performed in this order in the right thigh and right arm, respectively. Subsequently, an hour after the commencement of the resting period, the same procedure (i.e., resting, SFA FMD, and BA FMD) was repeated to determine the intra-day variability (visit 1 pre vs. visit 1 post). The same protocol was repeated during visit 2 and the inter-day variability was estimated based on first and second visits FMD measurements (visit 1 pre vs. visit 2). The FMD protocols were always performed first in the SFA then in the BA to ensure consistency across measurements and visits. The FMD in the SFA was performed first to maximize the volunteer’s collaboration as this is a more challenging procedure for the volunteer than the FMD in the BA. The two study sessions were organized at the same time of the day for each volunteer to eliminate any potential effect that may be associated with circadian rhythm and hormonal release (Etsuda et al. 1999; Mohd Azmi et al. 2021; Otto et al. 2004).

### Ultrasound assessment of arterial function

Endothelial-dependent vasodilation of the SFA and BA was assessed using FMD in accordance with recent guidelines (Thijssen et al. 2019). Arterial diameter and blood velocity of the SFA and BA were non-invasively assessed by means of a high-resolution duplex ultrasound device (Terason uSmart 3300, Teratech Corporation, Burlington, MA, USA) with a 15–4 MHz linear array transducer (Terason 15L4 Smart Mark, Teratech Corporation, Burlington, MA, USA), using a set frequency of 4.5 MHz. Pulse-wave Doppler signal was corrected at an insonation angle of 60°, and the sample volume (size of 1.5 mm) was placed at the centre of the arterial lumen. The right SFA was located and scanned longitudinally between 10 and 20 cm distal to the inguinal crease. A manual blood pressure cuff was positioned around the distal end of right thigh (3–4 cm proximal to the patella), distal to the imaged artery. Our FMD protocol focused on imaging the SFA specifically (rather than the common femoral artery, for example) with the cuff placed around the thigh because this was the only protocol that has been published that was demonstrated to be NO-mediated (Kooijman et al. 2008). The right BA was located and scanned longitudinally between 5 and 10 cm proximal to the antecubital fossa. A manual blood pressure cuff (different from the cuff used during SFA FMD) was positioned around the right forearm (about 2 cm distal to the antecubital fossa), distal to the imaged artery. In both the SFA and BA FMD protocols, once a satisfactory image of the artery was obtained (with clear vascular boundaries), the ultrasound probe was stabilized by means of an adjustable stereotactic probe-holding tool (FMD-probe-holder-xyz, Quipu S.r.l., Pisa, Italy). Arterial diameter and blood velocity were continuously recorded for 1 min (baseline), 5 min during which the cuff was inflated and maintained at a pressure of 220 mmHg, and 5 remaining minutes following the rapid cuff deflation (less than 3 s). The total time of the protocol was 11 min. The location of the transducer on the participant’s skin was marked and recorded to ensure consistency in placement during subsequent FMD measurements. All the FMD protocols were performed by a trained PhD student which, prior to the commencement of the present study, underwent a 4-month training in ultrasound imaging and FMD protocol by FMD trained researcher (CR) and also supported by a trained sonographer specifically for SFA imaging (MB). This consisted of six–eight supervised ultrasound scans per week of both the BA and SFA until the researcher could perform measurements independently. This was followed by approximately 80 unsupervised BA and SFA FMD protocols.

### Data analysis

Measurements of the arterial diameter and blood velocity were analyzed offline using an automated edge-detection software (Cardiovascular Suite, Quipu S.r.l., Pisa, Italy). All video recordings were analyzed by the same researcher (AD) who performed the FMD measurements. Video recordings were analyzed multiple times; each time, the region of interest—the portion of the image where the diameter is calculated—was moved to a different location across the artery image. This procedure was performed until three similar and reliable values were obtained. Baseline diameter was defined as the average diameter recorded during the minute preceding cuff inflation. Peak diameter was defined as the largest diameter observed following cuff deflation. Both parameters were then used to calculate the percent change in arterial diameter (i.e., FMD [%]) as described in the following formula:

$${\text{FMD}}\left(\text{\%}\right)\text{ = }\frac{\text{Peak Diameter}-\text{Baseline Diameter}}{\text{Baseline Diameter}}\times 100$$

Baseline values of shear rate, an adequate surrogate measure of shear stress (Pyke and Tschakovsky 2005), was calculated using the formula:

$$\text{Shear Rate}=\frac{4\times \text{Baseline Mean Positive Blood Velocity}}{\text{Baseline Mean Diameter}}$$

For FMD, arterial diameter, blood velocity, and shear rate, intra-day and inter-day reproducibility measurements were assessed using the coefficient of variation (CV), intraclass correlation coefficient (ICC), and mean absolute error (MAE).

The CV (%) were calculated on measurements obtained at visit 1 (visit 1 pre vs. visit 1 post) for the intra-day variability, and between visits (visit 1 pre vs. visit 2) for the inter-day variability, using the formula:

$${\text{CV}}\left(\text{\%}\right)={\frac{\text{Standard Deviation}}{\text{Mean}}} {\times} {100} $$

The lower the CV (%) reported, the lower the variability between assessments (higher reproducibility). Specifically, the quality of reproducibility of vascular measurements was classified as previously described: excellent (0–10%), good (10–20%), moderate (20–30%), and poor (> 30%) (van Mil et al. 2016). CV were estimated to assess both intra-day and inter-day variability of outcome measures of interest. Paired sample t tests were used to determine differences across CV of the dependent variables (i.e., resting diameter [mm], resting velocity [cm∙s^−1^], resting shear rate [s^−1^], and FMD [%]).

The ICC has a value between 0 and 1, referring to correlations within a class of data (Liljequist et al. 2019). The interpretation of ICC—as a measure of reliability—depends on how far the ICC number is from 1. Specifically: a value below 0.5 indicates poor reliability; a value between 0.5 and 0.75 indicates moderate reliability; a value between 0.75 and 0.9 indicates good reliability; a value above 0.9 indicates excellent reliability. Intraclass correlation coefficient estimates and their 95% confidence intervals were calculated based on a mean-rating (two measurements), absolute agreement, and two-way mixed-effects model (Koo and Li 2016).

Mean absolute error was used as a measure of the average magnitude of the absolute errors between paired measurements within the same dataset. The calculations of MAE were performed using on online calculator (AgriMetSoft, 2019).

Bland–Altman plots were also produced to evaluate measurement bias and agreement between FMD measurements (taken in different arteries and time points), by plotting the ‘bias’ (mean difference between paired FMD measurements: *visit 1 pre vs. visit 1 post, for intra-day; visit 1 pre vs. visit 2, for inter-day*) and the 95% limits of agreement (bias ± 1.96 × standard deviation of difference). The presentation of the 95% limits of agreement allows for a visual judgment of how well two measurements agree. The smaller the range between these two limits, the better the agreement is. Proportional bias was assessed via linear regression analysis (difference and mean as dependent and independent variables), and fixed bias was assessed by performing one-sample t test (mean difference vs. 0). Bland–Altman plots, in combination with ICC and CV, provide a more comprehensive evaluation of the reproducibility and reliability of outcome measurements.

### Statistical analysis

Independent sample t test were used to determine differences in participants baseline characteristics between groups (i.e., males vs. females). Two-way Repeated Measures ANOVA, with ‘Time (visit 1 pre, visit 1 post, visit 2)’ and ‘Artery (brachial, femoral)’ as within-subjects factors, was used to determine significant differences in resting diameter (mm), resting velocity (cm·s^−1^), resting shear rate (s^−1^), and FMD (%). Two-way Repeated Measures ANOVA, with ‘Time (intra-day, inter-day)’ and ‘Artery (brachial, femoral)’ as within-subjects factors, was used to determine significant differences in the CVs of resting diameter (mm), resting velocity (cm·s^−1^), resting shear rate (s^−1^), and FMD (%). Pearson correlation coefficients were determined for measuring the strength and direction of the linear relationship between paired variables, specifically, FMD and diameter of the BA and SFA (bivariate correlations; 2-tailed *p* value). For two females, it was not possible to collect reliable data for visit 2 due to some unavoidable limb movements occurred during the FMD protocols. Sample size estimation was performed using G*Power software (version 3.1.9.3) assuming an effect size *f* = 0.35, (given the lack of prior studies) and considering power of 0.80 and an alpha of 0.05, a sample size of *N* = 15 was estimated to detect a meaningful difference in CV between the SFA and BA (based on a two-way Repeated Measures ANOVA with ‘Time’ and ‘Artery’ as main within-subjects factors). The data in the tables and figures are presented as mean ± standard deviation (SD). A *p* value less than 0.05 was considered statistically significant. All statistical analyses were performed using statistical software IBM SPSS Statistics for Windows, version 28.0.1 (IBM Corp., Armonk, New York, USA).

## Results

### Study participants

Participants characteristics are summarized in Table 1. Participants were aged 24.7 (± 3.1) years old, with a healthy body mass index, 23.9 (± 2.7) kg·m^−2^. Significant differences in height, weight and age were observed between males and females. In both brachial and femoral arteries, males displayed larger resting artery diameter (mm) and lower FMD (%) than females (Table 1).

**Table 1:** Participants baseline characteristics

	Males	Females	Total
*N*	9	6	15
Age (yr.)	26.0 ± 3.1 *	22.8 ± 2.1	24.7 ± 3.1
Height (m)	1.78 ± 0.08 *	1.64 ± 0.06	1.73 ± 0.10
Weight (kg)	77.1 ± 13.5 *	63.3 ± 6.5	71.6 ± 13
BMI (kg·m^−2^)	24.1 ± 2.5	23.6 ± 3.2	23.9 ± 2.7
BA resting diameter (mm)	4.3 ± 0.5 **	2.9 ± 0.3	3.7 ± 0.8
BA resting velocity (cm·s^−1^)	19.0 ± 8.6	12.5 ± 1.9	16.4 ± 7.4
BA resting shear rate (s^−1^)	175.6 ± 80.2	177.6 ± 42.4	176.4 ± 65.7
BA FMD (%)	5.6 ± 1.9 *	8.5 ± 2.8	6.7 ± 2.6
SFA resting diameter (mm)	6.6 ± 0.5 **	5.5 ± 0.4	6.1 ± 0.8
SFA resting velocity (cm·s^−1^)	14.1 ± 2.4	13.2 ± 2.3	13.8 ± 2.3
SFA resting shear rate (s^−1^)	85.4 ± 15.2	97.4 ± 11.5	90.2 ± 14.7
SFA FMD (%)	3.6 ± 1.5 *	6.0 ± 1.1	4.6 ± 1.8

Data are presented as mean ± SD

*BA* brachial artery, *BMI* body mass index, *FMD* flow-mediated dilation, *SFA* superficial femoral artery

*Denotes significant difference (*p* < 0.05) between males and females within the same parameter

**Denotes significant difference (*p* < 0.001) between males and females within the same parameter

### Vascular parameters: FMD, velocity, shear rate, and arterial diameter

Data for resting diameter (mm), resting velocity (cm·s^−1^), resting shear rate (s^−1^), and FMD (%) assessed in the BA and SFA across different time points (i.e., visit 1 pre, visit 1 post, and visit 2), are reported in Table 2.

**Table 2:** Vascular parameters of the brachial and superficial femoral artery

Artery	Vascular parameter	Visit 1 (pre)	Visit 1 (post)	Visit 2
BA	Resting diameter (mm)	3.7 ± 0.8 #	3.7 ± 0.9 #	4.0 ± 0.8 #
	Resting velocity (cm·s^−1^)	16.4 ± 7.4 *	11.7 ± 4.6	17.9 ± 6.7
	Resting shear rate (s^−1^)	176.4 ± 65.7 *#	129.0 ± 44.8 #	189.8 ± 79.9 #
	FMD (%)	6.7 ± 2.6 #	6.6 ± 2.5 #	6.9 ± 2.3 #
SFA	Resting diameter (mm)	6.1 ± 0.8	6.2 ± 0.8	6.3 ± 0.8
	Resting velocity (cm∙s^−1^)	13.8 ± 2.3	13.3 ± 2.9	15.2 ± 3.1
	Resting shear rate (s^−1^)	90.2 ± 14.7	87.2 ± 21.9	98.4 ± 20.7
	FMD (%)	4.6 ± 1.8	4.8 ± 1.6	3.7 ± 2.2

Data are presented as mean ± SD

*BA* brachial artery, *FMD* flow-mediated dilation, *SFA* superficial femoral artery

*Denotes significant difference (*p* < 0.001) between ‘visit 1 pre’ and ‘visit 1 post’ within the same parameter and artery. #Denotes significant difference (*p* < 0.05) between arteries within the same parameter and time condition

There were no significant differences in arterial diameter and FMD within day (intra-day) and between days (inter-day) for both femoral and brachial arteries. In each time condition, arterial diameter of the BA was significantly lower than that of the SFA (*p* < 0.05), while the FMD of the BA was significantly higher than that of the SFA (*p* < 0.05). On the contrary, there were no significant differences in resting velocity between arteries, but in the BA only, resting velocity significantly declined across time points during visit 1 (intra-day) (*p* < 0.05). In regard to resting shear rate, it was significantly lower in the SFA compared to the BA (*p* < 0.05). In addition, there was a significant decline in resting shear rate (*p* < 0.001) in the BA across time points during visit 1 (intra-day), but that was not observed in the SFA.

**Fig. 2:**
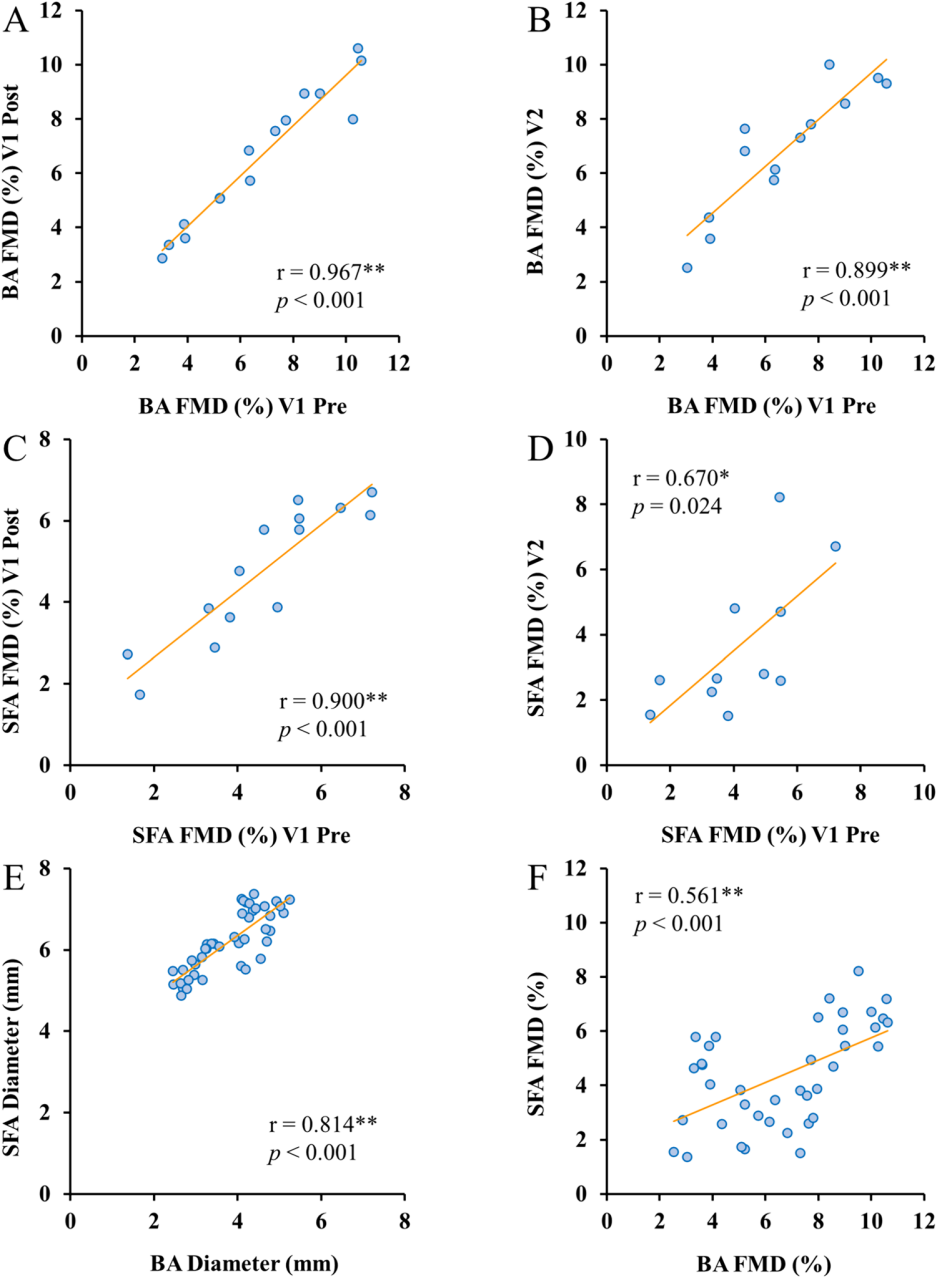
Correlations between different pairs of variables, specifically: (**A**) BA FMD (%) visit 1 pre vs. BA FMD (%) visit 1 post; (**B**) BA FMD (%) visit 1 pre vs. BA FMD (%) visit 2; (**C**) SFA FMD (%) visit 1 pre vs. SFA FMD (%) visit 1 post; (**D**) SFA FMD (%) visit 1 pre vs. SFA FMD (%) visit 2; (**E**) BA resting diameter (mm) vs. SFA resting diameter (mm); (**F**) BA FMD (%) vs. SFA FMD (%). **Denotes a significant correlation at the 0.01 level (2-tailed). *Denotes a significant correlation at the 0.05 level (2-tailed).* BA* brachial artery, *FMD* flow-mediated dilation, *SFA* superficial femoral artery, *V1* visit 1, *V2* visit 2

As reported in Fig. 2, BA FMD measurements were significantly highly correlated within the same day (*r* = 0.967, *p* < 0.001) and between days (*r* = 0.899, *p* < 0.001) (Fig. 2A, B, respectively). For SFA FMD, there was also a significant, strong positive correlation between assessments within the same day (r = 0.900, *p* < 0.001), while although significant, the positive correlation between days was only moderate (r = 0.670, *p* = 0.024) (Fig. 2C, D, respectively). Resting diameters were also highly correlated between both arteries (*r* = 0.814, *p* < 0.001) (Fig. 2E). FMD were significantly, but only moderately correlated between BA and SFA (*r* = 0.561, *p* < 0.001) (Fig. 2F).

### Intra-day and inter-day variability

Intra-day and inter-day CV, intraclass correlation coefficient (ICC), and mean absolute error (MAE) of the vascular parameters (i.e., resting diameter [mm], resting velocity [cm∙s^−1^], resting shear rate [s^−1^], and FMD [%]), measured in the BA and SFA, are reported in Table 3.

**Table 3:** Intra-day and inter-day coefficients of variation, intraclass correlation coefficients, and mean absolute errors of the brachial and superficial femoral artery

Artery	Vascular parameter	*N*	Intra-day CV (%)	ICC	MAE	*N*	Inter-day CV (%)	ICC	MAE
BA	Resting diameter (mm)	15	1.6 * (M: 1.6; F: 1.6)	0.993	0.1	13	3 (M: 3.1; F: 2.6)	0.970	0.2
	Resting velocity (cm∙s^−1^)	15	24.0 # (M: 22.3; F: 26.5)	0.568	4.9	13	24.7 # (M: 26.0; F: 21.7)	0.205	6.5
	Resting shear rate (s^−1^)	15	23.2 # (M: 22; F: 25)	0.494	52.2	13	22.5 # (M: 23.8; F: 19.5)	0.278	62.1
	FMD (%)	15	4.2 # (M: 3.8; F: 4.9)	0.967	0.4	13	8.7 # (M: 9.5; F: 6.8)	0.903	0.8
SFA	Resting diameter (mm)	15	1.9 (M: 1.4; F: 2.6)	0.967	0.2	13	1.9 (M: 2; F: 1.7)	0.967	0.2
	Resting velocity (cm∙s^−1^)	15	8.8 (M: 10.0; F: 7.0)	0.682	1.7	13	8.4 (M: 9.7; F: 5.4)	0.555	1.8
	Resting shear rate (s^−1^)	15	10.4 (M: 10.8; F: 9.7)	0.665	12.9	13	9.1 (M: 9.4; F: 8.4)	0.585	12.7
	FMD (%)	14	11.6 (M: 12.4; F: 10.5)	0.898	0.7	11	26.7 (M: 27.6; F: 24.3)	0.651	1.4

*BA* brachial artery, *CV* coefficient of variation, *F* females, *FMD* flow-mediated dilation, *ICC* intraclass correlation coefficient, *M* males, *MAE* mean absolute error, *SFA* superficial femoral artery

*Denotes significant difference (*p* < 0.05) between intra-day and inter-day within the same parameter and artery

#Denotes significant difference (*p* < 0.05) between arteries within the same parameter and intra/inter-day

BA resting diameter CVs (intra-day: 1.6%; inter-day: 3%) and ICCs (intra-day: 0.993; inter-day: 0.970) are indicative of excellent reproducibility and reliability, based on recent classification systems (Koo and Li 2016; van Mil et al. 2016). Excellent reproducibility and reliability were also found for SFA resting diameter (CV: 1.9%, ICC: 0.967 for both intra-and inter-day). BA resting velocity CVs (intra-day: 24.0%; inter-day: 24.7%) and ICCs (intra-day: 0.568; inter-day: 0.205) are indicative of moderate reproducibility and poor reliability, while for SFA, resting velocity had CVs (intra-day: 8.8%; inter-day: 8.4%) and ICCs (intra-day: 0.682; inter-day: 0.555) indicative of excellent reproducibility and moderate reliability. BA resting shear rate CVs (intra-day: 23.2%; inter-day: 22.5%) and ICCs (intra-day: 0.494; inter-day: 0.278) are indicative of moderate reproducibility and poor reliability, while for SFA, resting shear rate had CVs (intra-day: 10.4%; inter-day: 9.1%) and ICCs (intra-day: 0.665; inter-day: 0.585) indicative of good reproducibility and moderate reliability. Finally, BA FMD CVs (intra-day: 4.2%; inter-day: 8.7%) and ICCs (intra-day: 0.967; inter-day: 0.903) indicated excellent reproducibility and reliability, while SFA FMD CVs (intra-day: 11.6%; inter-day: 26.7%) and ICCs (intra-day: 0.898; inter-day: 0.651) showed good to moderate reproducibility and reliability.

**Fig. 3:**
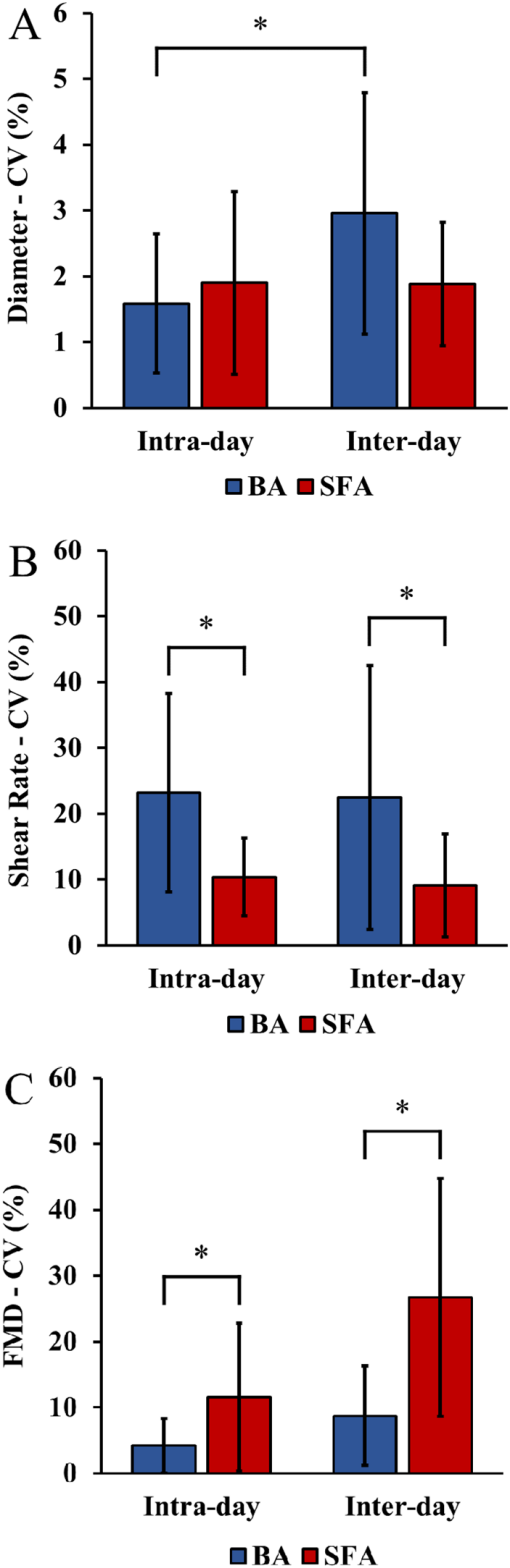
Intra-day and inter-day coefficients of variation of the brachial and superficial femoral artery. The vascular parameters presented are: (**A**) baseline diameter (mm); (**B**) baseline shear rate (s^−1^); (**C**) FMD (%). Data are presented as mean ± SD. *Denotes significant difference (*p* < 0.05). *BA* brachial artery, *CV* coefficient of variation, *FMD* flow-mediated dilation, *SFA* superficial femoral artery

Inter-day CV for BA resting diameter was significantly higher than intra-day CV (*p* < 0.05); but no significant differences between intra-day and inter-day were detected for SFA (Fig. 3A). No significant differences between intra-day and inter-day CV were detected for resting shear rate or FMD in both arteries (Fig. 3B, C). No significant differences between arteries were detected within and between days for resting diameter. Both intra-day and inter-day CV for resting velocity were significantly higher for BA (CV: 24.0%, 24.7%, respectively) than SFA (CV: 8.8%, 8.4%, respectively) (*p* < 0.05). Similarly, for resting shear rate, both intra-day and inter-day CV were significantly higher for BA (CV: 23.2%, 22.5%, respectively) than SFA (CV: 10.4%, 9.1%, respectively) (*p* < 0.05) (Fig. 3B). Both intra-day and inter-day CV for FMD were significantly lower (CV: 4.2%, 8.7%, respectively) than SFA (CV: 11.6%, 26.7%, respectively) (*p* < 0.05) (Fig. 3C). Individual data points depicting males and females are reported in the supplementary material (Fig. 5).

MAE for BA FMD (%) were 0.4% FMD for intra-day, and 0.8% FMD for inter-day, which are equivalent to 6% and 12% error in relation to their absolute average (for intra-day and inter-day, respectively). Estimated MAE for the SFA FMD (%) were higher, with 0.7% FMD for intra-day, and 1.4% FMD for inter-day, which is equivalent to 15% and 34% error in relation to their absolute average. MAE for resting diameter were more similar between arteries (e.g., inter-day: 0.2 mm). MAE for BA resting velocity were 4.9 cm∙s^−1^ and 6.5 cm·s^−1^ for intra-day and inter-day, respectively, while for SFA these values were lower (MAE intra-day: 1.7 cm·s^−1^; inter-day: 1.8 cm·s^−1^). Finally, MAE for BA resting shear rate were 52.2 s^−1^ and 62.1 s^−1^ for intra-day and inter-day, respectively, and as was observed for the velocity measures, MAE for the SFA were lower (MAE intra-day: 12.9 s^−1^; inter-day: 12.7 s^−1^).

### Measurement bias: Bland–Altman analysis

**Fig. 4:**
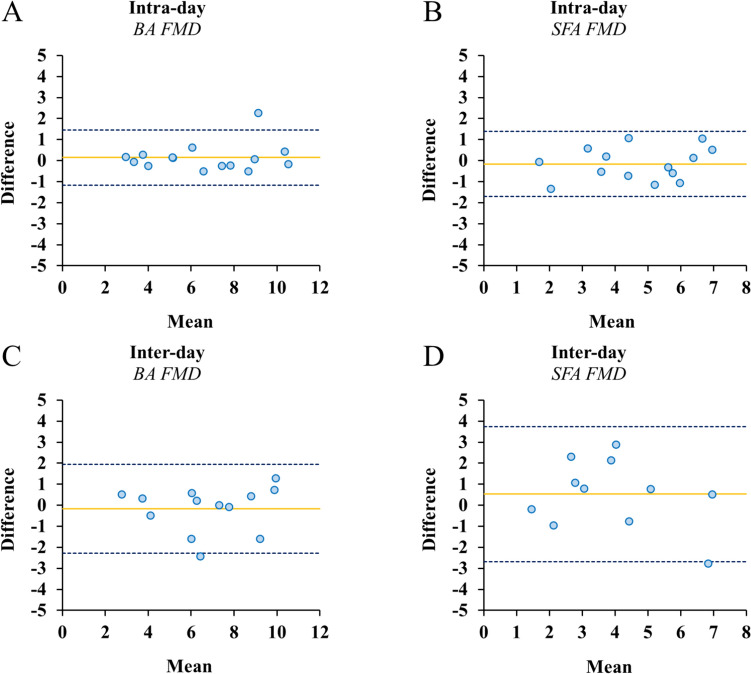
Bland–Altman plots of intra-day and inter-day variability of FMD, specifically: (**A**) BA FMD intra-day; (**B**) SFA FMD intra-day; (**C**) BA FMD inter-day; (**D**) SFA FMD inter-day. The x-axis shows the mean FMD across time points and the y-axis shows the difference between the two paired FMD measurements. The dashed lines represent the 95% limits of agreement; the continuous line represents the bias. *BA* brachial artery, *FMD* flow-mediated dilation, *SFA* superficial femoral artery

The intra-day and inter-day variability of FMD (%) estimated in the BA and SFA are also represented on Bland–Altman plots (Fig. 4), specifically displaying the difference between the two paired FMD measurements on the y axis and the mean FMD across time points on the x axis. In regard to intra-day variability, the 95% limits of agreement for BA FMD are –1.2 and 1.5, while for SFA FMD are –1.7 and 1.4 (Fig. 4A, B, respectively). The mean difference (bias) of the intra-day FMD measurements for the BA and SFA are 0.15 and –0.16, respectively. In regard to the inter-day variability, the 95% limits of agreement for BA FMD are –2.3 and 2.0, while for SFA FMD are –2.7 and 3.7 (Fig. 4C, D, respectively). The bias of the inter-day FMD measurements for the BA and SFA are –0.16 and 0.53, respectively. Linear regression analysis revealed no proportional bias regarding intra-day BA (β: 0.16; *p* = 0.57) and SFA FMD (β: 0.22; *p* = 0.45), and inter-day BA (β: 0.10; *p* = 0.74) and SFA FMD (β: –0.29; *p* = 0.39). In addition, one-sample t test showed no fixed bias regarding intra-day BA (*p* = 0.41) and SFA FMD (*p* = 0.46), and inter-day BA (*p* = 0.60) and SFA FMD (*p* = 0.31).

## Discussion

The present study compared, for the first time, the intra-day and inter-day variability of FMD and related parameters (blood velocity, shear rate, arterial diameter) between the upper limb (BA) and lower limb (SFA) conduit arteries in young healthy adults. We have observed that both BA and SFA FMDs reported moderate to excellent levels of reproducibility and reliability, as assessed by both CV and ICC. This is further supported by Bland–Altman analysis, with no fixed bias and no proportional bias between measurements detected. Although no significant differences in FMD values were detected within and between days for both arteries, FMD in the BA was significantly more reproducible (4.2%; 8.7%) than in the SFA (11.6%; 26.7%, respectively for intra-and inter-day). Resting blood velocity and shear rate were less reproducible in the BA in comparison to the SFA, while arterial diameter had similar reproducibility in both arteries. Overall, there were no significant differences in CV between intra-day and inter-day measures, except for BA resting diameter which had a significant improvement in CV for intra-day (1.6%) in comparison to inter-day (3%). Overall, these data indicate that greater caution must be taken when using FMD in the lower limbs, as the reproducibility/reliability of the measure is poorer than for brachial FMD, given an identical volume of ultrasonography training.

In agreement with previous studies, we report a higher FMD for the BA compared to the SFA (Climie et al. 2018; Nosova et al. 2014; Schreuder et al. 2014). Upper and lower limb vessels exhibit heterogeneous vascular responses in terms of both vasodilatory and vasoconstrictory responses (O’Brien et al. 2019; O’Brien and Shivgulam 2023) which is related to the size of conduit arteries in upper limbs (e.g., BA) being smaller than conduit arteries in lower limbs (e.g., SFA) (Thijssen et al. 2008). This not only affects the magnitude of the FMD response, but also the wall-to-lumen ratio. For example, individuals with enlarged wall-to-lumen ratio (typical of smaller arteries) displayed increased vasoactive responses due to more smooth muscle relative to elastic laminae (O’Brien and Shivgulam 2023).

The current study’s BA FMD CVs (intra-day: 4.2%; inter-day: 8.7%) and ICCs (intra-day: 0.967; inter-day: 0.903) indicate excellent reproducibility and reliability (Koo and Li 2016; van Mil et al. 2016), which were in line with the levels recommended by current FMD guidelines (Corretti et al. 2002; Thijssen et al. 2019). For example, Corretti et al. (2002) suggested that a sonographer should have a CV for consecutive FMD scans below 25% (which corresponds to approximately an absolute difference of 2–3% in FMD, assuming a baseline FMD of 10%). More recent recommendations suggest a stricter CV below 15% (Thijssen et al. 2019). It is important to acknowledge that there is a wide variability of reported reproducibility among studies for BA FMD, with inter-day CVs ranging from 1.8% (Sorensen et al. 1995) to 50.3% (De Roos et al. 2003). Nonetheless, the majority of studies assessing brachial FMD reproducibility in a sample of healthy adults report CVs of approximately 9% and 13% for intra-day and inter-day, respectively (e.g., Craiem et al. 2007; Ghiadoni et al. 2012), which are consistent with the CVs reported in the current study.

Similarly, for BA resting diameter, CVs (intra-day: 1.6%; inter-day: 3%) and ICCs (intra-day: 0.993; inter-day: 0.970) indicate excellent reproducibility and reliability (Koo and Li 2016; van Mil et al. 2016), and are consistent with levels recommended by the FMD guidelines (CV < 2% for arterial diameter) (Thijssen et al. 2019). Furthermore, the levels of reproducibility for BA diameter also align with other studies, specifically with CV around 1.6% and 3.4% for intra-day and inter-day, respectively (Donald et al. 2008; Hijmering et al. 2001), and ICC of 0.968 and 0.948 for intra-day and inter-day, respectively (Meirelles et al. 2007). In contrast, BA shear rate measures revealed moderate reproducibility and poor reliability, as demonstrated by higher CVs (intra-day: 23.2%; inter-day: 22.5%) and lower ICCs (intra-day: 0.494; inter-day: 0.278) (Koo and Li 2016; van Mil et al. 2016). Contrary to diameter and FMD, there are no guidelines established for resting shear rate, and no reports of reproducibility of this measure in other studies. The high variability in brachial resting blood velocity (inter-day CV: 24.7%), is likely the key contributor to the variability observed in shear rate (inter-day CV: 22.5%), especially given that the arterial diameter is relatively stable. This is further confirmed by the observation that BA velocity and shear rate are highly correlated (*r* = 0.848, *p* < 0.001). This was also observed for the SFA (*r* = 0.802, *p* < 0.001), with similar CVs observed across shear rate (inter-day: 9.1%) and velocity (inter-day CV: 8.5%). However, it is currently uncertain why a higher variability in shear rate is observed for BA compared to SFA, but we have confirmed these differences in subsequent data collection (data not shown). This is perhaps to be expected given that blood velocity in the BA is a parameter that can change dramatically with little stimuli, as for instance, even a change in posture (standing, as compared to supine/seated posture) can impact measures of blood velocity within this artery (Newcomer et al. 2008).

Moreover, a key element to consider is how much the observed variability in resting artery diameter and shear rate might influence reproducibility of the FMD response. This has not been fully established in the literature to date, but several studies report that FMD is inversely correlated with arterial diameter (Herrington et al. 2001; Silber et al. 2001; Thijssen et al. 2008). In addition, diameter is also inversely correlated to post-occlusion shear rate (Pyke and Tschakovsky 2005), which directly drives the FMD response (Gibbs et al. 2011; Pyke and Tschakovsky 2005). The low levels of variability in diameter measurements observed in the present study suggest that variations in arterial diameter may not have contributed significantly to variations in FMD in the BA. However, we cannot exclude the possibility that variability in shear rate may have contributed to variations seen in BA FMD. We anticipate that variability in resting shear rate would translate into post-occlusion shear rate variability, which would influence FMD (post-occlusion shear rate data could not be reliably collected as part of the present study). For example, Parkhurst et al. (2012) have found a positive (low) correlation between resting shear rate and FMD (*r* = 0.43), indicating that reductions in shear rate would result in proportional reductions in FMD.

In the SFA, FMD measures had CVs (intra-day: 11.6%; inter-day: 26.7%) and ICCs (intra-day: 0.898; inter-day: 0.651) indicative of good to moderate reproducibility/reliability (Koo and Li 2016; van Mil et al. 2016). Although, our SFA FMD intra-day CV is below 15%, and our inter-day CV is slightly above 25%, which is near the levels of reproducibility recommended (Corretti et al. 2002; Thijssen et al. 2019), it clearly indicates poorer reproducibility in comparison to BA FMD. Unfortunately, a direct comparison with the literature is challenging, as most studies do not report reproducibility in the SFA (Bapir et al. 2022b; Ratcliffe et al. 2017). In agreement with our findings, McLay et al. (2016) showed that FMD reproducibility in the lower limb popliteal artery is also highly variable as indicated by high CV (intra-day: 44%; inter-day 40%) and low ICC (intra-day: 0.36; inter-day 0.25) (McLay et al. 2016). To the best of our knowledge, this is the only study assessing FMD reproducibility, and specifically reporting CV and ICC. A recent study by Bapir et al. (2022b) reported Bland–Altman analysis, showing no bias for common femoral FMD, however, no objective measures of reproducibility and reliability were reported.

The fact that lower limb arteries appear to have a poorer level of repeatability for FMD compared to BA FMD, given the same amount of operator training, implies that caution should be taken when using the technique in research studies, particularly if reproducibility of the protocol is not assessed. This might be especially critical for smaller effect sizes, for example when the desired detectable difference in FMD is ≤ 1% FMD. In that case, for both cross over and parallel designs an absolute difference in FMD CVs of at least 10% (note that the absolute difference between femoral and brachial inter-day CV was 18% in the current study) equate to more substantial discrepancies in the number of volunteers needed to reach the desired effect size, which might lead to underpowered studies (e.g., Charakida et al. 2013). These differences in reproducibility between arteries can be more easily interpreted when comparing their inter-day MAE for BA (0.8%) and SFA (1.4%) with the clinically relevant threshold of 1% FMD, typically associated with a 13% increased risk of a future CVD event (Inaba et al. 2010). While there are several studies using SFA FMD as the primary outcome (for example, prolonged strenuous exercise (Dawson et al. 2008), uninterrupted sitting (Ballard et al. 2017; Thosar et al. 2015, 2014) and implementation of sit-to-stand desks (Bodker et al. 2021)), that report changes in SFA FMD of more than 2%, more subtle, yet clinically relevant effect, (e.g., nutritional interventions) may not be easily detected with the variability observed in SFA.

Understanding what is contributing toward an increased variability in SFA FMD is a challenging question. The variability in both SFA diameter (CVs of 1.9%, for both intra-day and inter-day) and SFA resting shear rate (CVs for intra-day: 10.4; inter-day: 9.1) indicate very good reproducibility and reliability (Koo and Li 2016; van Mil et al. 2016). For example, shear rate variability in the SFA is significantly lower than in the BA, despite higher variability on SFA FMD. Together, this indicates that variability in diameter and shear rate alone are not likely to explain the increased variability in the SFA compared to the BA.

Importantly, there are several technical aspects that might help explain the poorer reliability of SFA FMD. For example, the location of the cuff in the distal third of the thigh (just above the patella) might be contributing to participant discomfort during inflation—as suggested by Bapir et al. (2022b)—which can lead to extra movements through the period of ischemia. Furthermore, the fact that the cuff is located at a distance of only ~10 cm from the imaged artery results in involuntary movement of the skin and consequently the probe, during inflation. This creates extra uncertainty in repositioning the probe to the location pre-ischemia, likely generating additional error. Applying the cuff in the calf (a few cm below the patella), rather than in the thigh, has been shown to be more comfortable for participants (Bapir et al. 2022b) and as the distance between imaged artery and cuff is higher, we anticipate that this might also reduce movement of the skin/probe. In addition, some studies (Bapir et al. 2022a, 2022b) perform FMD in the common femoral, which also results in a higher distance between imaged artery and cuff. However, the only FMD protocol in the lower limb arteries that has been shown to be NO-mediated is performed in the SFA, with the cuff placed on the thigh (Kooijman et al. 2008) and this is also the most widely used in the literature (e.g., Ballard et al. 2017; Caldwell et al. 2020; Carter et al. 2019; de Groot et al. 2005; Heinen et al. 2015; Hundley et al. 2007; Naylor et al. 2021; Schreuder et al. 2014; Thosar et al. 2015, 2014; Walker et al. 2019). The vasodilation of the common femoral artery has not been shown to be NO-mediated, and as endothelial NO synthase content is heterogeneous throughout the arterial tree (Laughlin et al. 2003), the relative contribution of NO to FMD in the superficial versus common femoral artery may differ. In summary, the distance between imaged artery and cuff might be an important aspect to take into consideration when deciding on a FMD protocol in future studies, but careful consideration must be taken if NO-dependency is a target mechanism.

Intrinsically, sonographer technical competency is a substantial source of variability that plays a critical role in the reproducibility of ultrasound-related measures, particularly FMD. Prior to the commencement of the present study, the sonographer performed more than 80 BA and SFA FMD protocols. While this volume of training was clearly sufficient to reach excellent levels of reproducibility in the BA, this was not the case for SFA. Interestingly, further training in the SFA FMD protocol (which occurred after the current study was completed), resulted in an improved inter-day FMD CV of 8.9%, with similar levels for diameter (CV = 2.8%) and shear rate (CV = 10.9%) (data not shown). The further training performed by the operator was not designed as a ‘controlled intervention’, (not included in the current study), but it was mainly driven by our own group’s necessity to improve the reliability of the measure to perform our own studies. Regardless, our own experiences support the fact that extra training can reduce the variability of the FMD in the SFA to similar levels as BA. However, future studies should systematically address how much more training is needed in the SFA to ensure reproducible measures.

From a technical point of view, there are also modifications that could be implemented in future that might improve the repeatability of the technique. For example, we (and others) use an adjustable stereotactic probe-holding tool to secure the ultrasound probe in place. While this works well in the BA, in the SFA it made re-capturing the image post-occlusion more challenging for the operator, due to the extra volume of movement. As such, we recommend that operators train the SFA FMD protocol by hand-holding the probe to be able to compensate for that movement. However, hand-holding the probe might require further training to reach adequate competency (Thijssen et al. 2019). Alternatively or concurrently, changing the location of the cuff to the calf, is also likely to reduce movement and discomfort of participants.

Generally, and as recommended by current FMD guidelines, we attempted to control for intake of caffeine, alcohol, medication, exercise, menstrual cycle, and time of the day, as much as we could. For example, female participants performed the testing sessions within two consecutive days, to minimize the effects of their monthly hormonal variation on FMD outcomes. Although, inter-day CVs tended to be higher than intra-day CVs for both BA and SFA FMD (BA: 8.7% vs. 4.2%; SFA: 26.7% vs.11.6%), this was not statistically significant. We interpret this as further evidence that conditions were kept consistent enough between days in our FMD protocols. Similarly, other studies have not reported any significant differences in CV between intra-day and inter-day measures for both FMD and arterial diameter (Ghiadoni et al. 2012; Meirelles et al. 2007; Onkelinx et al. 2012). Overall, this indicates that if environmental/participant conditions are well-controlled, the time (intra/inter-day) between measurements may only marginally affect FMD, when assessing short-term reproducibility. It is, however, important to consider, that if the duration of research studies is long, medium-term (e.g., weeks/months) and long-term (e.g., months/years) reproducibility must be re-assessed, as this is likely to be affected (Charakida et al. 2013).

One of the main limitations of the present study is that we performed our measurements over two independent days rather than three, which would have given us more robust and reliable assessments of reproducibility. Unfortunately, due to limitations on volunteers’ availability, this was not viable. Other studies that have performed these assessments over 3 separate occasions report similar reproducibility of FMD in the BA to that which was observed here with 2 sessions. Secondly, we would have preferred to assess inter-day variability in females across the same stage of the menstrual cycle in consecutive menstrual cycles, but due to time restrictions, we opted for measuring inter-day repeatability over 2 consecutive days to minimize hormonal changes. It would have been informative to estimate shear rate area under the curve to assess whether variability in shear rate stimulus post-deflation would significantly affect FMD variability. However, the collection of this set of data proved difficult in the SFA due to substantial movement of the artery during deflation. Future studies should explore this further. The use of a probe holder might have been a limiting factor in assessing FMD in the SFA. While this works well in the BA, future studies may consider hand-holding the probe to assess FMD in lower limb arteries. Finally, the different time interval separating visits among male and female participants may be a further limitation. Although we do not detect significant differences in variability between males and females (data analysis not shown), the current study was not powered to assess sex differences. Future studies should address this question by comparing males and females using a standardized time interval (e.g., 4 weeks). Furthermore, it is important to highlight that we cannot exclude the possibility that longer time intervals between visits could result in higher variability, meaning that the values reported in our study could be an underestimation.

## Conclusion

To the best of our knowledge, the present study is the first to directly compare the reproducibility and reliability of ultrasound-related measures on upper and lower limb conduit arteries in young healthy adults. Our data indicate that FMD in the SFA is less reproducible than in the BA, given identical volume of ultrasound training; this is of relevance for the increasing number of studies that use both FMD assessments to test the impact/efficacy of interventions, such as nutrition/diet (Bapir et al. 2022a), prolonged sitting (Climie et al. 2018; Restaino et al. 2015; Thosar et al. 2014), bed rest (Bleeker et al. 2005; Nosova et al. 2014), and step reduction (Boyle et al. 2013). Future studies should take into consideration that higher volume of training might be needed for a typical researcher to ensure adequate levels of FMD reproducibility in the lower limb conduit arteries, as well as adjustments to the protocol that may facilitate that process. Overall, it is important that levels of reproducibility/reliability are assessed prior to the commencement of studies to ensure adequate estimation of sample size and statistical power. These should also be reported in the literature when FMD protocols are being used in published studies. Future studies should focus on: (i) determining the ideal protocol to minimize random error for FMD in the lower limbs; (ii) determining what volume of additional training is needed to systematically reach acceptable levels of repeatability, and (iii) considering how data analysis might also contribute to the reproducibility of the technique.

## Supplementary Information

Below is the link to the electronic supplementary material.

Supplementary file1 (DOCX 167 KB)

## Data Availability

The data that support the findings of this study are available on request from the corresponding author, [CR].
