# Supplementary material for: Variability of flow-mediated dilation across lower and upper limb conduit arteries
Source: Eur J Appl Physiol. 2024 Jun 15;124(11):3265–78. doi: 10.1007/s00421-024-05517-z (PMC11519148; doi:10.1007/s00421-024-05517-z)
Supplement: Supplementary file 1 — Supplementary file1 (DOCX 167 KB) [file 421_2024_5517_MOESM1_ESM.docx]

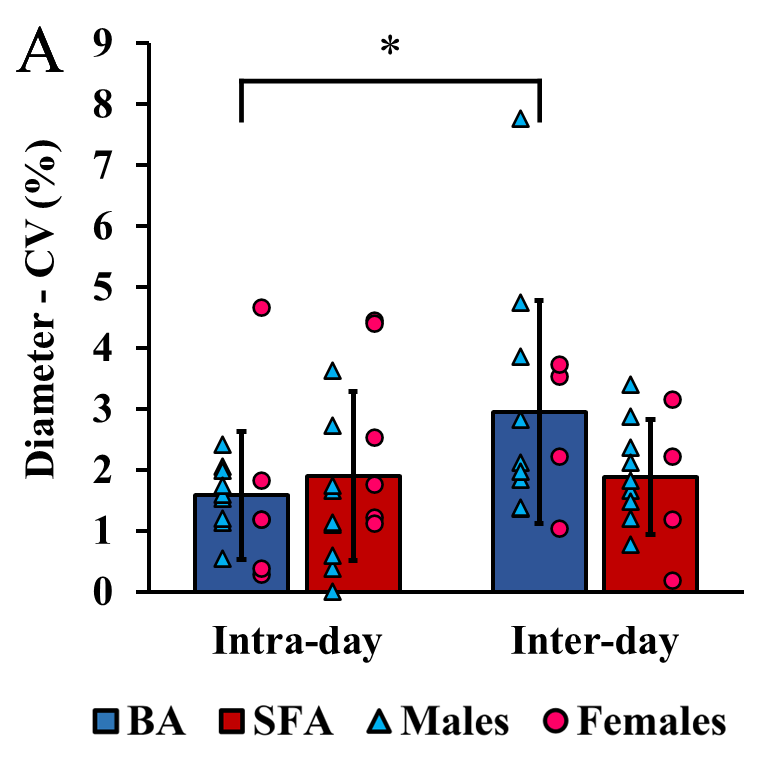


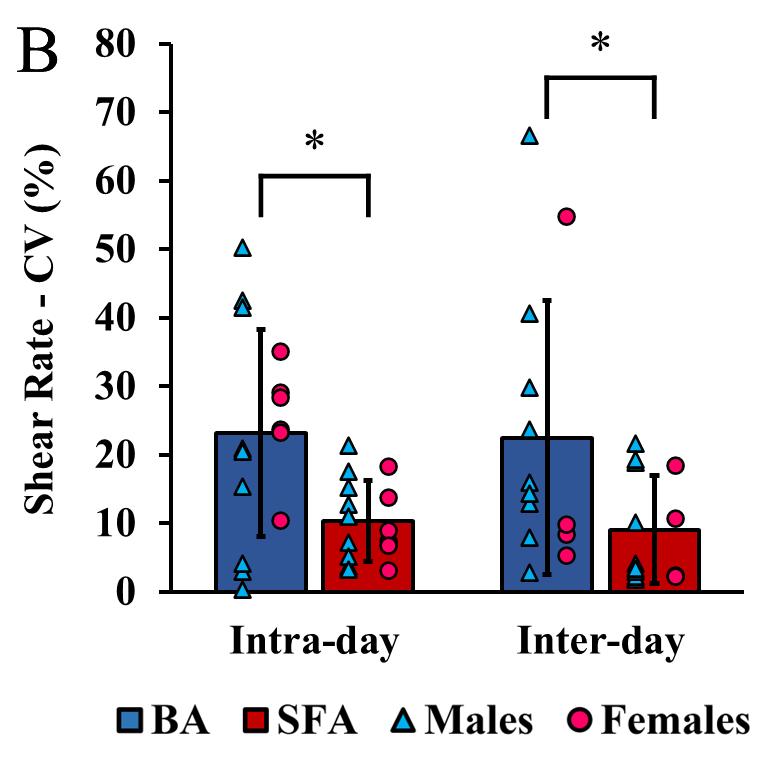


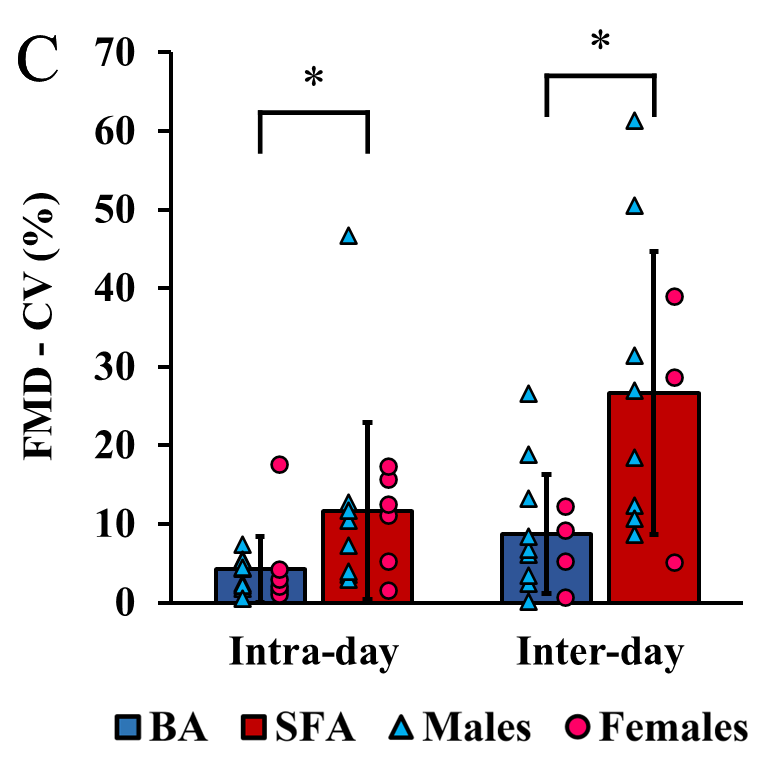


**Figure 5.** Intra-day and inter-day coefficients of variation of the brachial and superficial femoral artery, with individual data points depicting males and females. The vascular parameters presented are: (A) baseline diameter (mm); (B) baseline shear rate (s^−1^); (C) FMD (%). Data are presented as mean ± SD. * denotes significant difference (*p* < 0.05). *BA, brachial artery; CV, coefficient of variation; FMD, flow-mediated dilation; SFA, superficial femoral artery.*
